# Designing Nanoconjugates to Effectively Target Pancreatic Cancer Cells *In Vitro* and *In Vivo*

**DOI:** 10.1371/journal.pone.0020347

**Published:** 2011-06-27

**Authors:** Jameel Ahmad Khan, Rachel A. Kudgus, Annamaria Szabolcs, Shamit Dutta, Enfeng Wang, Sheng Cao, Geoffry L. Curran, Vijay Shah, Steven Curley, Debabrata Mukhopadhyay, J. David Robertson, Resham Bhattacharya, Priyabrata Mukherjee

**Affiliations:** 1 Department of Biochemistry and Molecular Biology, College of Medicine, Mayo Clinic, Rochester, Minnesota, United States of America; 2 Department of Medicine and Physiology, College of Medicine, Mayo Clinic, Rochester, Minnesota, United States of America; 3 Neuroscience Research, College of Medicine, Mayo Clinic, Rochester, Minnesota, United States of America; 4 Department of Surgical Oncology, University of Texas MD Anderson Cancer Center, Houston, Texas, United States of America; 5 Cancer Center, College of Medicine, Mayo Clinic, Rochester, Minnesota, United States of America; 6 Department of Chemistry and University of Missouri Research Reactor, University of Missouri, Columbia, Missouri, United States of America; 7 Department of Biomedical Engineering, College of Medicine, Mayo Clinic, Rochester, Minnesota, United States of America; University of Pennsylvania, United States of America

## Abstract

**Background:**

Pancreatic cancer is the fourth leading cause of cancer related deaths in America. Monoclonal antibodies are a viable treatment option for inhibiting cancer growth. Tumor specific drug delivery could be achieved utilizing these monoclonal antibodies as targeting agents. This type of designer therapeutic is evolving and with the use of gold nanoparticles it is a promising approach to selectively deliver chemotherapeutics to malignant cells.

Gold nanoparticles (GNPs) are showing extreme promise in current medicinal research. GNPs have been shown to non-invasively kill tumor cells by hyperthermia using radiofrequency. They have also been implemented as early detection agents due to their unique X-ray contrast properties; success was revealed with clear delineation of blood capillaries in a preclinical model by CT (computer tomography). The fundamental parameters for intelligent design of nanoconjugates are on the forefront. The goal of this study is to define the necessary design parameters to successfully target pancreatic cancer cells.

**Methodology/Principal Findings:**

The nanoconjugates described in this study were characterized with various physico-chemical techniques. We demonstrate that the number of cetuximab molecules (targeting agent) on a GNP, the hydrodynamic size of the nanoconjugates, available reactive surface area and the ability of the nanoconjugates to sequester EGFR (epidermal growth factor receptor), all play critical roles in effectively targeting tumor cells *in vitro* and *in vivo* in an orthotopic model of pancreatic cancer.

**Conclusion:**

Our results suggest the specific targeting of tumor cells depends on a number of crucial components 1) targeting agent to nanoparticle ratio 2) availability of reactive surface area on the nanoparticle 3) ability of the nanoconjugate to bind the target and 4) hydrodynamic diameter of the nanoconjugate. We believe this study will help define the design parameters for formulating better strategies for specifically targeting tumors with nanoparticle conjugates.

## Introduction

Cancer claims nearly 25% of deaths annually. Pancreatic cancer is the fourth leading cause of cancer related deaths in America, in both men and women. Despite vast efforts to detect and treat pancreatic cancer, the incidence and mortality rates remain virtually the same. Early diagnosis and efficient delivery of therapeutic agents to malignant cells remain the two major challenges in cancer management strategies [1].

Monoclonal antibodies against growth factor receptors have been shown to be viable treatments for inhibiting cancer growth [2]. Utilizing these monoclonal antibodies as targeting agents for tumor specific delivery is evolving as a promising approach to selectively deliver chemotherapeutics [3]. Inorganic nanomaterials are being studied as the delivery vehicle for targeted drug delivery. Gold nanomaterials are of particular interest due to the unique physico-chemical and optoelectronic properties, ease of synthesis and surface modification [1], [4], [5], [6], [7], [8], [9], [10], [11], [12], [13].

Gold nanoparticles (GNPs) have recently been used to kill tumor cells by hyperthermia using non-invasive radiofrequency [14]. Their utility as a contrast agent has also been demonstrated by clear delineation of blood capillaries in a preclinical model by CT (computer tomography) in comparison to the conventional iodine based contrast agents [15]. Both studies are hopeful and their utility is further encouraged by the safety profile [16], [17].

Epidermal growth factor receptor (EGFR) is an important target in cancer research. It is overexpressed in a number of human malignancies including pancreatic cancer. Human EGFR is a transmembrane glycoprotein [3], [18], [19], [20]. It consists of an extracellular ligand binding domain, a hydrophobic transmembrane domain and an intracellular tyrosine kinase domain. Ligand binding to EGFR induces receptor homo/heterodimerization leading to the phosphorylation of tyrosine residues. Phosphorylation of EGFR activates complex down stream signaling events leading to proliferation, migration, invasion, and inhibition of apoptosis of cancer cells [21], [22], [23].

The monoclonal anti-EGFR antibody, Cetuximab (C225), is a unique targeting agent to target EGFR-positive cancer cells. Cetuximab was approved by the FDA for the treatment of patients with EGFR positive colorectal cancer (CRC) [21], [22], [23]. It has also been either approved or is in different phases of clinical trials in many other malignancies such as NSCLC (non small cell lung carcinoma), SCCHN (squamous cell carcinoma of the head and neck) and pancreatic cancer [24], [25], [26]. Cetuximab is a chimeric human∶murine immunoglobulin G1 (IgG1) monoclonal antibody (MoAb). The binding of C225 to EGFR leads to receptor internalization and degradation without receptor phosphorylation, thus inhibiting EGFR-associated pathways [24], [25], [26].

Despite the emerging utility of GNPs in targeted delivery fundamental questions remain unanswered. What are the design criteria for fabricating nanoconjugates that will ensure maximum uptake in cancer cells? Herein, we utilize cetuximab (C225) as a targeting agent and GNP as a model system. We demonstrate that the number of C225 antibodies on a GNP (C225∶GNP ratio), the hydrodynamic size, the available reactive surface area and the ability of the nanoconjugate to sequester EGFR, all play critical roles in effectively targeting tumor cells *in vitro* and *in vivo* in an orthotopic model of pancreatic cancer. Uptake studies with the isotype control, GNP-IgG, indicate that the specificity of tumor cell targeting is dependent on the nanoparticle surface coverage by C225. Non-specific uptake decreases when the C225 to GNP ratio increases. These studies are critical to develop an efficient targeted delivery system for future clinical use.

The work presented herein shows the systematic characterization of GNP-C225 nanoconjugates. The specific targeting potential was investigated, both *in vitro* and *in vivo* in an orthotopic model of pancreatic cancer. Our results demonstrate that the hydrodynamic radius, accessible reactive surface area and loading capacity of C225 on GNPs play critical roles for efficient targeting of tumor cells. These findings highlight key parameters to be considered for a promising nanoparticle based drug delivery system for future clinical application.

## Results

### Characterization of the nanoconjugates with variable antibody to nanoparticle ratios

In order to understand the parameters required for specific targeting of nanoconjugates to tumor cells, we selected cetuximab (C225) as a targeting agent. C225 is a chimeric human-murine monoclonal antibody that binds to the extracellular domain of EGFR [3]. EGFR is overexpressed in a number of human malignancies including pancreatic cancer, rendering it an attractive target [18]. To determine the optimum valency of C225 on GNPs (the number of C225 molecules per GNP and hence C225∶GNP ratio) for intracellular uptake, we synthesized various nanoconjugates with multiple C225∶GNP ratios. Characterization of these nanoconjugates was performed using several physicochemical techniques: UV-Visible (UV-Vis) spectroscopy, transmission electron microscopy (TEM), dynamic light scattering (DLS) and radioiodination of C225 with ^125^I. The GNPs used in this study were synthesized by sodium borohydride reduction of tetrachloroaurate [27], [28]. As previously reported, the presence of a surface plasmon resonance band at ca. 510 nm confirms the formation of spherical gold nanoparticles (Figure S1). The formation of spherical GNPs and their 5 nm size diameter was further confirmed by TEM (data not shown).

**Figure 1:**
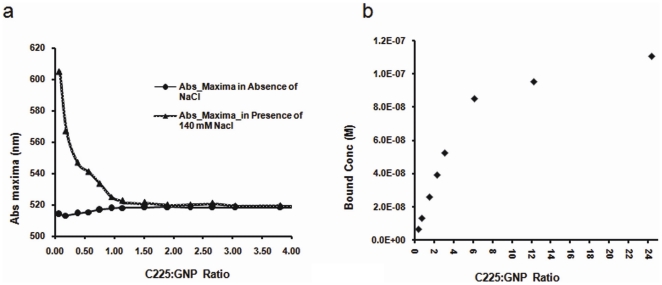
Determination of binding capacity and stability of GNP-C225 conjugates. **a.** Change in absorption maxima as a function of increasing concentration of C225 in absence (spheres) and presence of NaCl (triangles). **b.** Concentration of C225 bound to GNP as a function of increasing concentration of C225, determined by measuring the concentration of free C225 labeled with I125 in the supernatant, after pelleting down the GNP-C225 conjugates by ultracentrifugation.

The GNP-C225 conjugates were then synthesized using this naked GNP solution and purified as described in the materials and methods section. The antibody spontaneously binds to the GNPs through Au-S and Au-N bonding [19]. The production of GNP-C225 was monitored by UV-Vis spectroscopy. It is evident that with the addition of C225 there is a gradual red shift in the SPR band of the naked gold, from 510 nm to 519 nm (Figure 1a). Such a red shift in the SPR band of the GNPs suggests the perturbation of the electrical double layer by the antibody surrounding the GNPs and thus indicates binding of the antibody to the nanoparticles [29], [30].

To further confirm the GNP-C225 conjugation we challenged the nanoconjugates against salt induced aggregation (critical coagulation concentration). Addition of 140 mM NaCl has been reported to result in aggregation of naked or partially covered particles, such aggregation leads to a dramatic red shift in the SPR band. The absorption spectra of the nanoconjugates were recorded 15 minutes after incubation with 140 mM of sodium chloride (NaCl). As expected, the naked GNPs showed a drastic red shift in the SPR band from 510 nm to around 600 nm (Figure 1a) confirming aggregation of uncovered nanoparticles [31]. Salt induced aggregation was directly related to the increased loading of C225 on the GNP surface, hence the SPR band completely disappeared at a C225 to GNP ratio of 3 (three C225 molecules per GNP) suggesting the absence of available reactive surface to salt induced aggregation.

### Quantification of C225 binding to gold nanoparticles by radioiodination

The binding of C225 to GNPs was quantified using radiolabelled ^125^I-C225. A fixed concentration of GNPs was incubated with ^125^I-C225 at various C225∶GNP ratios. The conjugates were centrifuged at high speed (45000 g) for 1 hour to separate the unbound antibody from the nanoconjugates. The efficiency of the GNP-C225-I^125^ binding was then determined by measuring the emission of gamma rays emanating from the free and bound ^125^I-C225 in the supernatant and the pellet fractions, respectively. As shown in Figure 1b and Figure S2, there was a very high binding percentage of C225 (∼90%) to GNPs up to a ratio of 3 (3 C225 molecules per GNP). However, a minimal increase in binding is observed above a ratio of 3. This small increase at high antibody concentrations might be due to weak nonspecific protein-protein interactions between the bound and free antibody. This data further corroborates the observations made by UV-Vis spectroscopy discussed above; where the extent of salt induced aggregation was found to be minimal at a ratio of 3.

### Targeted delivery of gold nanoparticles to pancreatic cancer cells in vitro

**Figure 2:**
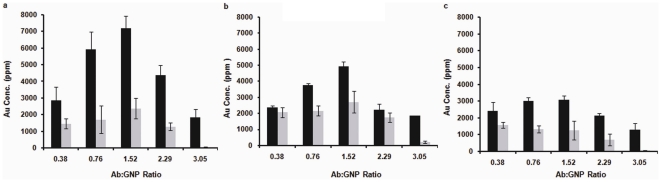
Uptake of GNP-Ab conjugates by pancreatic cancer cells. **a.** AsPC-1 Cells, **b.** PANC-1 cells, **c.** MiaPaca-2 cells, treated with GNP-C225 (black bars) or GNP-IgG (grey bars) prepared at different Ab∶GNP ratios. Y axis represents Gold concentration as ppm of total dry mass of cells.

To understand the role of multivalency on the intracellular uptake by human pancreatic cancer cell lines with variable EGFR expression, we treated AsPC-1, PANC-1 and MiaPaca-2 cells with GNP-C225 or nonspecific isotype control GNP-IgG at different antibody to GNP (Ab∶GNP) ratios. EGFR expression in these cells follows the order AsPC-1≥PANC-1>MiaPaca-2 [19]. The amount of cellular uptake for GNP-C225 and GNP-IgG by the different cell lines was quantified by determining the gold content in the cells through instrumental neutron activation analysis (INAA). Figures 2a, 2b and 2c represent the gold content of AsPC-1, PANC-1 and MiaPaca-2 cells, respectively. It is evident from the figures that the uptake of the nanoconjugates in all three cell lines gradually increases with the increasing C225∶GNP ratio (increased valency). The maximum uptake was observed with the 1.5 ratio (black bars); beyond this ratio the uptake of the nanoconjugates gradually decreased. The uptake trend of the nanoconjugates follows a similar pattern in all three cells lines. It is also important to note that the extent of uptake was much less for the corresponding ratios for isotype controls, GNP-IgG (grey bars). Interestingly, the non-specific uptake of GNP-IgG gradually decreased with increasing IgG∶GNP ratio where minimum uptake was observed at the ratio of 3. It is likely that at the lower Ab∶GNP ratio, the available free reactive surface area on the GNP can non-specifically bind either to the serum proteins or cell membranes or both; which in turn could promote the non-specific uptake of the nanoconjugates. Adsorption of serum components is known to influence the uptake of GNP [32]. Increasing Ab∶GNP ratios should increase the antibody coverage of the nanoparticle surface, thus providing less open surface area available for non-specific interactions. Reduction of such non-specific interactions might explain reduced uptake of GNP-IgG conjugates observed at higher ratios (Figure 2).

### Internalization of the nanoconjugates by pancreatic cancer cells in vitro

**Figure 3:**
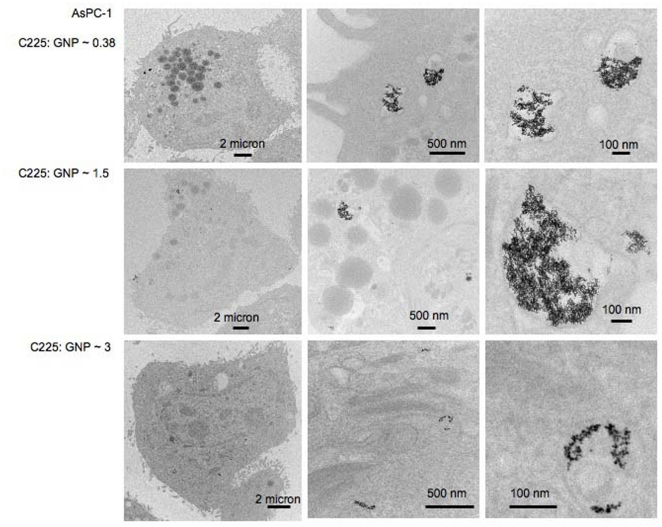
Internalization of different GNP-C225 conjugates by AsPC-1 Cells. TEM images at different magnifications, showing internalization of GNP-C225. Please see Figures S3 and S4 for TEM images illustrating GNP-C225 internalization by PANC-1 and MiaPaca-2 cells.

To further confirm the intracellular uptake and localization of GNPs, TEM analysis was performed after treating the cells with the same nanoconjugates used for the uptake studies. Figure 3 shows TEM micrographs of AsPC-1 cells after treatment with GNP-C225 at varying C225∶GNP ratios. Low, medium and high magnification images are represented for C225∶GNPs ratios of 0.38, 1.5 and 3.0 (Figure 3). The nanoconjugates are shown within the vesicular structures of the cells, confirming intracellular uptake of the nanoconjugates. Similar results were also obtained with PANC-1 and MiaPaca-2 cells (Figures S3, S4). The differential uptake of the GNPs by the cancer cells having different C225∶GNP ratios might also be due to the differential affinity of the nanoconjugates to bind EGFR, or size of the nanoconjugates. Interestingly, when the same cell lines were treated with GNP-IgG, the isotype control, most of the GNPs are found at the periphery of the cell membrane with minimal endocytosis (Figure S5). This is consistent with a recent report describing the targeting of a solid tumor with transferrin coated GNPs [33].

### Hydrodynamic diameter of the nanoconjugates and their ability to sequester EGFR

**Figure 4:**
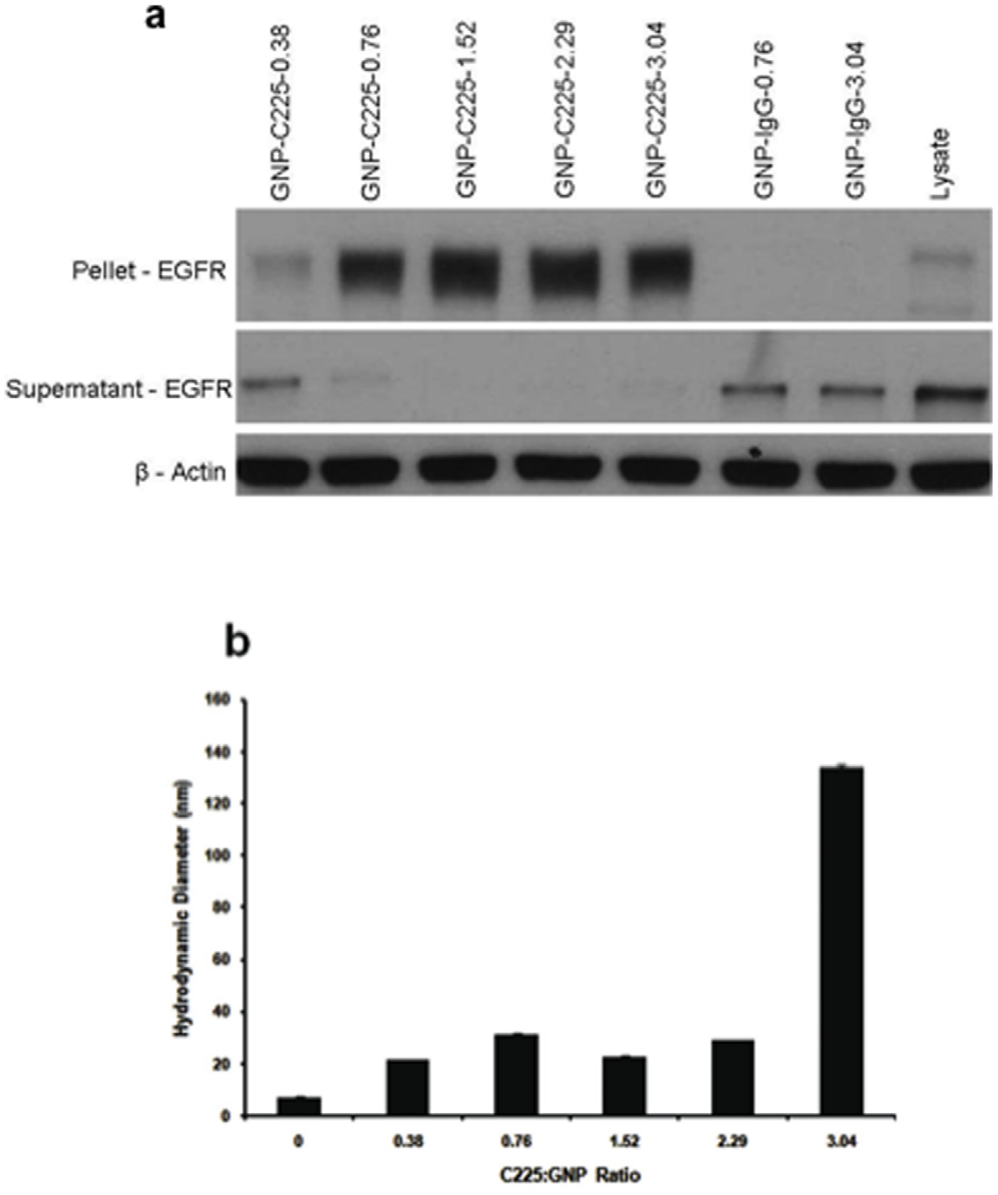
Characterization and in vitro uptake of GNP-C225. **a.** Binding of GNP-C225 and GNP-IgG to EGFR verified by Western Blot analysis. Cell lysates from AsPC-1 cells were incubated for 2 hrs at room temperature with GNP-C225 and GNP-IgG conjugates with various ratios of antibody on the surface of the particle. After incubation the samples were spun at high speed and the supernatant was collected and the pellet was washed once and respun; the nanoconjugate fraction was 20 times the concentration of the supernatant. The pellet and the supernatant fractions were loaded on a 7.5% SDS-PAGE gel and analyzed for EGFR. **b.** Hydrodynamic diameter of GNP-C225 measured by Dynamic Light Scattering Spectroscopy.

In an attempt to elicit the mechanism for differential uptake of GNP-C225, we hypothesized that the different C225∶GNP ratios possess differential ability to bind EGFR. To validate this hypothesis, we performed western blot analysis. The various GNP-C225 and GNP-IgG conjugates were incubated with AsPC-1 cell lysates for 2 h, then centrifuged at high speed. The pellet was washed once and then the supernatant and pellet were subjected to western blot analysis to detect the relative presence of EGFR in the different fractions. The results are shown in Figure 4a. This clearly demonstrates there is a progressive depletion of EGFR in the supernatant with an increasing C225∶GNP ratio (middle panel). Also interesting to note an enrichment of EGFR in the pellet fraction (upper panel) reaching a plateau at a ratio of 1.5–2.29, most likely a function of C225 loading on the pellet surface. β-actin levels were used as loading controls demonstrating equal loading of protein in different lanes. It is also important to note that when the same lysates were treated with GNP-IgG, hardly any detectable EGFR was found in the pellet fraction, mostly it was found in the supernatant fraction, further confirming the binding specificity of GNP-C225 to EGFR. Together, this data suggests that the GNP-C225 conjugates can specifically sequester EGFR from the cell lysates and the sequestering ability increases with an increasing number of C225 molecules on the GNP surface.

The ability of the nanoconjugates to sequester EGFR with a C225∶GNP ratio of 1.5 to 3 is similar; however, the *in vitro* intracellular uptake decreases as the ratio changes from 1.5 to 3. To explain the decreasing uptake of the nanoconjugates with an increasing C225∶GNP ratio, we investigated the role of the hydrodynamic diameter of the nanoconjugates. The hydrodynamic diameter (HD) of the conjugates was determined through dynamic light scattering spectroscopy (DLS) measurements (Figure 4b). Recently, HD has been shown to be critical for tumor targeting of quantum dots [34]. Furthermore, it has also been demonstrated that the *in vitro* cellular uptake and response is dependent on the size of the nanoconjugates. Upon conjugation with a 0.38 C225∶GNP ratio the HD of the GNPs immediately increases from 7 nm to 21 nm, further confirming the binding of C225 to the GNP surface [35]. The HD remains around 21 nm until a ratio of 2.29 then sharply increases at a ratio of 3. It should be noted from Figures 1b and S2 that nearly all antibody molecules remain bound to the GNP surface up to a ratio of 3. However, there is only a gradual increase in binding above this ratio, most likely attributed to nonspecific binding. This nonspecific binding of antibody molecules may be causing the formation of a loose secondary layer around GNP-C225 resulting in a sharp increase in the HD at higher C225∶GNP ratios. Such a sharp increase in HD could also be due to the heterogeneity of the sample. Obtaining a discrete number of antibody molecules per GNP is very difficult to achieve experimentally. The relationship between the multivalency of the nanoparticles to their hydrodynamic diameters needs to be further investigated. TEM analysis confirmed that the sharp increase in the HD is not due to aggregation of the nanoparticles. The TEM micrographs did not show any significant aggregation of the nanoparticles with different C225∶GNP ratios (Figure S6).

### Delivery of GNP-C225 in an orthotopic model of pancreatic cancer

**Figure 5:**
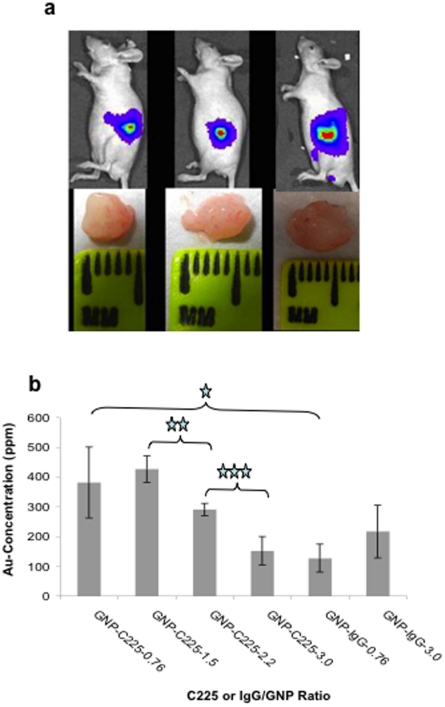
Characterization of AsPC-1 orthotopic tumor growth and in vivo Au uptake in the tumors. **a.** Bioluminescence images on day 12 depicting tumor presence (top panel) and digital camera images (bottom panel) of tumor masses excised after termination of the mice above. The mice were sacrificed on day 13, 24 hrs after nanoconjugate treatments. **b.** *In vivo tumor* uptake of GNP-C225 and GNP-IgG conjugates at varying ratios of antibody; 24 h after the intraperitoneal injection of the conjugates into an orthotopic model of pancreatic cancer. The uptake was determined by measuring the gold concentration in the tumors by INAA. Y axis represents gold concentration as ppm.

**Figure 6:**
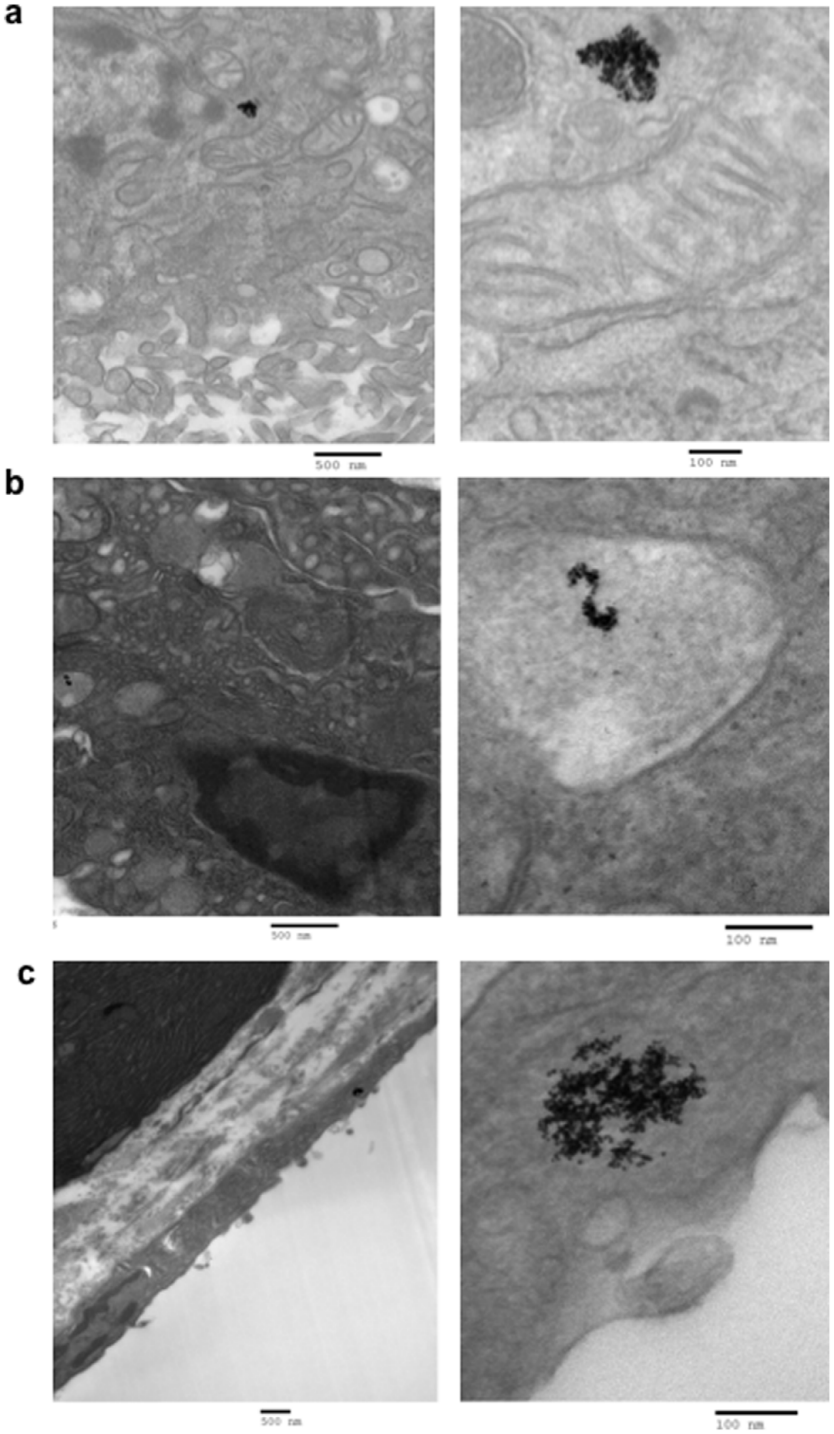
Representative TEM images of tumor sections illustrating nanoconjugate location within the tumor tissue. **a and b.** GNP-C225 with the 3.0 ratio of Ab∶GNP are shown on the left in a low magnification to show a large surface area of tumor tissue and on the right the magnification is increased to elucidate the internalization of the conjugates themselves. **c.** GNP-C225 with the 1.5 ratio of Ab∶GNP are shown on the left in low magnification to show a large surface area of tumor tissue and on the right the magnification is increased to reveal the conjugates themselves inside cellular space.

To further investigate the ability of these nanoconjugates to specifically target tumor cells *in vivo*, we utilized an orthotopic pancreatic cancer model. The model was generated after surgically implanting AsPC-1 cells expressing luciferase into the pancreas of nude mice and allowed to grow for 12 days. Every third day the mice were injected with 200 µl of ketamine and 100 µl of luciferin and imaged for tumor growth progression by bioluminiscence measurments. This orthotopic model tries to mimic the human pathogenesis of pancreatic cancer [36] and allows tumor cells to experience the effects of the microenvironment in the pancreas. On day 12, GNP-C225 and GNP-IgG were administered i.p. to study tumor uptake and distribution of nanoconjugates having different Ab∶GNP ratios. The mice were sacrificed 24 h after nanoconjugate injection and the tumors and other vital organs were collected for determination of gold content by INAA. Figure 5a shows a representative example of a bioluminescence image of mice 12 days after orthotopic implantation of the tumor cells into the pancreas (upper panel). Strong bioluminescence suggests the formation and growth of a tumor in the pancreas. Tumor growth was further confirmed by measuring the tumor size at the end of the experiments after sacrificing the mice (lower panel). The tumor size measured using the formula ab^2^/2 (a = length, b = width) was ∼62 mm^3^ when nanoconjugates were administered for biodistribution studies. Figure 5b shows the tumor uptake of GNP-C225 and GNP-IgG at varying Ab∶GNP ratios as a function of gold content determined by INAA. The *in vivo* and *in vitro* tumor uptake of the nanoconjugates followed a similar pattern. Statistical analysis using the two-tailed students t-test demonstrated that the uptake of GNP-C225 with a ratio of 0.76 and 1.5 were significantly higher than the ratio of 3 (p<0.05). Furthermore, the uptake of the GNP-C225 conjugates was significantly higher (p<0.05) than the isotype control, GNP-IgG at all ratios except 3. As mentioned earlier, the HD of GNP-C225 remained constant from a ratio of 0.38 to 2.29 (around 21 nm) but dramatically increased at a ratio of 3 (134 nm). Theoretically, the larger HD of the nanoconjugates at a ratio of 3 could contribute to lower diffusion and in turn reduction in uptake [37]. This illustrates that the HD is a critical factor for *in vivo* uptake and consequently a key factor for active targeting. To further investigate the intratumoral (inside the cells; or in the vasculature or in the capsule outside of the tumor tissue) location of the nanoconjugates, we performed TEM on the tumor tissues shown in figure 5a, which were obtained after sacrificing the mice 24 h post intraperitoneal administration of different nanoconjugates. Figure 6 shows the location of GNPs in different areas of the tumor tissues in case of GNP-C225 (C225∶GNP 3.0 (Figure 6a and b) and 1.5 (Figure 6c) treated groups. It is evident that GNPs are inside the cells (Figure 6a, left and right panels are images of the same area, different magnification) as well as the capsule covering the tumor tissue (Figure 6b, left and right panel are images of the same area, different magnification). However, the nanoconjugates are mostly localized outside of the tumor tissue in case of the GNP-IgG treated group (IgG∶GNP 1.5) (Figure S7).

The presence of injected conjugates in other major organs was also determined by INAA (Figure S8). Gold uptake was also found in other organs, such as spleen, liver, lung, kidney and brain [38], [39].

## Discussion

Targeted delivery of inorganic nanomaterials is an essential area of research for nanomedicine. Unique physicochemical properties of inorganic nanomaterials may be utilized for several biomedical applications such as detection/diagnosis, therapy and imaging. Thus, it is important to define the design parameters to specifically deliver nanoconjugates to the cells of interest. There are several key factors that may define the success of targeted delivery; (i) selection of an appropriate model to study the delivery approach; (ii) selection of an effective targeting agent; (iii) optimization of the number of targeting agents per nanoparticle; (iv) availability of free reactive area on the particle surface that may initiate non-specific binding; (v) ability of the targeting agent to sequester the target and (vi) hydrodynamic size of the nanoconjugates.

Most of the delivery strategies are tested in non-orthotopic models where tumors are generated away from the original site. A major advantage to this model is tumors are easy to develop and growth can be monitored by manual measurement with slide calipers. A disadvantage of this model is that the tumor cells do not experience a true tumor microenvironment. In contrast, the orthotopic model implants tumor cells in the organ of tumor origin, rendering it a more realistic model. Thus, we selected the orthotopic model to study the design parameters necessary for nanoconjugates to successfully and specifically target a tumor.

We selected cetuximab as a targeting agent and gold nanoparticles as the scaffold for targeting delivery. One of the major advantages to utilizing gold nanoparticles is their formation of stable bonds with biomolecules such as organothiols, organoamines, proteins, and antibodies [5]. We demonstrated the significant binding of cetuximab to GNPs up to a ratio of 3. (C225∶GNP = 3) beyond which only a marginal increase in binding is observed. This marginal increase might be due to the lack of free reactive surface area remaining on the GNPs. The nature of bonding between gold and proteins has been the subject of intense investigation over the last several decades. It is now generally accepted that there are three main types of interactions that occur between a protein/antibody and a gold nanoparticle; (i) electrostatic interactions of negatively charged GNPs with positively charged proteins; (ii) covalent interactions between the thiol/amine groups present within amino acid residues in antibodies and the GNPs; and (iii) hydrophobic interactions between proteins and GNPs [19], [40]. Using X-ray photoelectron spectroscopy and thermogravimetric analysis we previously demonstrated that cetuximab utilizes thiol and amine functionalities to bind to the surface of gold nanoparticles [19], [40].

It is also imperative to validate the design parameters for different cell lines with variable expression of the target receptor. This is particularly important considering the heterogeneous nature of tumors. In these studies we employed three different pancreatic cancer cell lines with variable expression of EGFR. We also demonstrated that intracellular uptake of GNP-C225 is not only dependent on the number of cetuximab molecules, but also depends on their ability to bind EGFR, as well as the hydrodynamic size of the nanoconjugates. It is also evident that non-specific uptake can be minimized *in vitro* by optimizing the coverage of the targeting agent on the nanoparticle surface. Maximum specific uptake of GNP-C225 is observed when there are three C225 molecules on a GNP even though the overall uptake is reduced. This data further demonstrates the combined importance of the number of targeting agents per particle and the hydrodynamic size of the nanoconjugates. This is also reflected in the difference between the specific uptake of GNP-C225 and the non-specific uptake of GNP-IgG.

Recently, several groups have been focusing on understanding the design parameters for efficiently targeting tumors with nanoconjugates in a preclinical model. It has also been reported that a nanoconjugate with a size of <100 nm is efficiently targeting tumor cells [41]. Antibody targeted liposomes have been found to exert enhanced efficacy over their non-targeted counterparts [42]. Furthermore, delivery of siRNA using transferring-targeted polymeric nanoparticles demonstrated better efficacy even though their kinetics, biodistribution and tumor uptake was similar [43]. Similar observations were also made in a study of transferrin-coated gold nanoparticles; it was demonstrated that ligand content on the nanoparticles are important for targeting [33]. Here, we explore an orthotopic model of pancreatic cancer and in turn, demonstrate the critical roles that the number of antibody molecules on the GNP, the ability of the nanoconjugates to bind the target receptor, the availability of the reactive surface for non-specific interaction and the hydrodynamic size of the nanoconjugates all play in efficient design.

Taken together our data suggests that the specific targeting of tumor cells depends on a number of important factors; 1) targeting agent to nanoparticle ratio; 2) availability of the reactive surface area on the nanoparticle; 3) ability of the nanoconjugate to bind the target and 4) hydrodynamic diameter of the nanoconjugate. Thus, intelligent design for successful targeting of tumor cells requires optimization of these crucial factors. We believe this study will help define the design parameters for formulating better strategies for specifically targeting tumors with nanoparticle conjugates.

## Materials and Methods

### Synthesis and characterization of GNP-antibody conjugates

The naked GNPs were synthesized by reduction of 100 ml of 0.1 mM HAuCl4 (Aldrich) solution with 50 ml of a freshly prepared aqueous solution containing 4.3 mg of sodium borohydride (Aldrich) under constant stirring, overnight at ambient temperature. The GNPs were characterized by transmission electron microscopy (TEM) after drop-coating 100 µl of the sample on a carbon-coated copper grid. The size of the nanoparticles was determined from analysis of the TEM images.

The GNP-antibody conjugates were synthesized by mixing the desired antibody (C225 or IgG) with the naked GNP solution. C225 is purchased in a solution of 2 mg/ml (ErbituxTM Injection, ImClone Inc and Bristol-Myers Squibb Co.) and human IgG was purchased in a solution of 10.0–11.2 mg/ml (Jackson Immuno Research Laboratories, Inc.). These solutions were diluted in water to a 1 ml final volume with the desired µgs present and then added drop wise to 100 ml of naked GNPs. This solution was stirred vigorously at ambient temperature for 2 hrs. The mixture was then centrifuged at 22,000 rpm in a Beckman Ultracentrifuge in a 50.2 Ti rotor to separate the desired GNP-antibody from unconjugated antibody. The conjugates formed a loose pellet at the bottom of centrifuge tube and were collected after careful removal of the supernatant. The gold concentration of the conjugates was determined from absorbances obtained by UV-Visible spectrometry (UV 2401PC, Shimadzu Corp) at 500 nm (A500), taken before and after centrifugation and by instrumental neutron activation analysis (INAA).

### Determining the number of C225 molecules bound to a GNP

Aliquots of C225 were labeled to a high specific activity using the chloramine-T procedure. 500 µg of C225 was diluted to a final volume of 200 µl with 100 mM phosphate buffer, pH 7.4. Then 1.0 mCi (10 µl) carrier free Iodine-125 radionuclide was added to the antibody solution. Immediately thereafter, 100 µg chloramine-T (100 µl of 10 µg/µl diluted in phosphate buffer, pH 7.4) was added and incubated for 2 minutes with intermittent vortexing. The reaction was quenched with the addition of 500 µg of sodium metabisulfite (100 µl of 50 µg/µl diluted in phosphate buffer, pH 7.4). The final product was dialyzed against PBS in a 1000 MWCO dialysis membrane (Spectra/Por 7) overnight at 4°C with two changes of 500 ml phosphate buffered saline pH 7.4. The specific activity of the labeled antibody was determined by the TCA (trichloroacetic acid) precipitation method. In brief, 5 µl of a 1∶200 dilution of the original labeled mixture was mixed with 100 µl of BSA and precipitated with 200 µl TCA. The mixture was centrifuged for 5 minutes at 15000 g. Radioactivity in the precipitate and supernatant were counted in a Packard gamma (Packard, Cobra D5003) counter. The labeled preparation was not used for further studies if the labeled antibody was <99%. The protein concentration in the labeled antibody stock was determined by the bicinchoninic acid (BCA) method.

Different amounts of labeled C225 were added to a solution of GNPs and incubated for 2 hours as described above. The conjugates were then centrifuged at 45000 g for 1 hour in a Beckman Ultra using a TL100.2 rotor. The radioactive emission from 10 µl of the pellet and supernatant was measured in a gamma counter as described above. The emission from the original labeled protein and the supernatant after centrifugation were used to calculate the concentration of C225 bound to GNP surface.

### Cell culture and treatments

Pancreatic cancer cell lines: AsPC-1, PANC-1 and MiaPaca-2 were grown in RPMI and Dulbecco's Modified Eagles medium (Gibco) supplemented with 10% fetal bovine serum (Gibco) and 1% antibiotics (Pen/Strep) at 37°C, in a humidified chamber under 20% O_2_ and 5% CO_2_ environment until confluent. The cells were incubated with different GNP-Ab conjugates at a final concentration of 50 µg/ml of gold for 2 hours followed by a rinse with PBS to remove non-bound conjugates. The cells were trypsinized and pelleted in a centrifuge at 1300 rpm for 5 minutes. The cell pellets were washed twice with PBS and analyzed for gold content by INAA or fixed in Trumps solution for TEM analysis.

### Measurement of gold content by Instrumental Neutron Activation Analysis (INAA)

Samples were analyzed by instrumental neutron activation analysis at the University of Missouri Research Reactor Center as previously described. Briefly, tissues and cell pellets were prepared by weighing the samples into high-density polyethylene irradiation vials and lyophilized to a dry weight. Solution samples were prepared by gravimetrically transferring 100 µl to an irradiation vial followed by lyophilization. All samples were loaded in polyethylene transfer “rabbits” in sets of nine and irradiated for 90 s in a thermal flux density of approximately 5×10^13^ n cm^−2^ s^−1^. The samples were then allowed to decay for 24 to 48 h and counted on a high-purity germanium detector for 3600 s at a sample-to-detector distance of approximately 5 cm. The mass of gold in each sample was quantified by measuring 411.8 keV gamma ray from the β^−^ decay of ^198^Au (t_1/2_ = 2.7 days). The area of this peak was determined by the Genie ESP spectroscopy package from Canberra. A minimum of six geometrically equivalent comparator standards were also run. The standards were prepared by aliquoting approximately 0.1 (n = 3) and 0.01 (n = 3) µg of gold from a (10.0±0.5) µg/mL certified standard solution (High-Purity Standards) in the polyethylene irradiation vials, and were used with each sample set.

### Dynamic Light Scattering Spectroscopy (DLS)

Determination of the hydrodynamic (HD) diameter of the GNP-C225 conjugates was determined by DLS in a Zetasizer instrument (Nano ZS, Malvern Instruments Ltd) equipped with a 633 nm LASER.

### Transmission electron microscopy (TEM)

TEM samples involving cells were prepared as described previously [29], [40]. In brief, cells were treated with gold nanoconjugates for 2 h in the presence of serum. After the incubation, cells were washed thrice in PBS and cell pellets collected after trypsinization and centrifugation at 1500 rpm for 5 minutes. The resultant cell pellets were further washed thrice with PBS, and fixed in Trumps fixative (1% glutaraldehyde and 4% formaldehyde in 0.1 M phosphate buffer, pH 7.2) and processed for TEM sectioning as previously described. Micrographs were taken on a TECNAI 12 operating at 120 KV.

TEM samples from the extracted mice tumors were prepared as follows; on day 13 the mice (n = 3) were sacrificed and the tumors were collected, weighed, measured and placed immediately in trumps fixative solution and stored on ice. The tumors were briefly removed from the trumps solution and sliced thinly for TEM mounting. Micrographs were taken on a TECNAI 12 operating at 120 KV.

### EGFR pull down/depletion assay

To determine the binding of the conjugates to EGFR, 100 µl of AsPC-1 cell lysates (containing 1.8 mg/ml of total protein) was incubated with 100 µl of 100 ug/ml GNP-C225 and GNP-IgG at various ratios of antibody for 2 hrs at ambient temperature. The mixtures were then centrifuged at 30,000 rpm for 1 h. The supernatant was removed and saved. The remaining pellet was washed with 100 µl of water and respun for 1 h at 30,000 rpm. The second supernatant was removed and saved. 20 µl of 2× SDS-PAGE loading dye was added to the pellet and 10 µl the initial supernatant was taken and 10 µl 2× SDS-PAGE loading dye was added. All samples were placed in a 95°C heating block for 5 minutes and subsequently loaded to a 7.5% SDS-PAGE gel. The gel was run for 45 mins at 150 V and then were transferred to a PVDF membrane and blotted for EGFR with an anti-EGFR antibody against its c-terminal region (Santa Cruz).

### Animal handling and in vivo tumor uptake

For the generation of orthotopic pancreatic tumor models, 1.5×10^6^ AsPC-1 cells were injected into the pancreas of nude mice. Every third day the mice were injected with 200 µl of ketamine and 100 µl of luciferin and imaged for tumor growth progression. Twelve days after tumor cell implantation, the mice were randomly distributed into 6 groups (n = 3). GNP-C225 and GNP-IgG conjugates at a gold concentration of 220 µg/ml was injected i. p. to each mouse. The mice were sacrificed 24 hrs after the injection and the tumor mass along with other organs and blood were collected and analyzed for gold content.

### Statistical analysis

Statistical analysis was done by a two-tailed student t-test and a value of P<0.05 was considered to be significant.

## Supporting Information

Figure S1 **Representative absorption spectrum of the GNP used in the study.**
(TIF)

Figure S2 **Binding of C225 to GNP determined by I^125^ labeled C225**: Concentration of C225 bound to GNP with increasing concentration of C225 represented as the fraction of total C225 added.
(TIF)

Figure S3 **Internalization of different C225-GNP conjugates by Panc-1 Cells.** Representative TEM images at different magnifications showing internalization of C225-GNP conjugates.
(TIF)

Figure S4 **Internalization of different C225-GNP conjugates by MiaPaca-2 Cells.** Representative TEM images at different magnifications showing internalization of C225-GNP conjugates.
(TIF)

Figure S5 **Internalization of GNP-IgG conjugates by PANC-1 and MiaPaca-2 Cells.** Representative TEM images at different magnifications showing internalization of GNP-IgG conjugates.
(TIF)

Figure S6 **TEM images of GNP-C225 conjugates synthesized at different C225∶GNP ratio.** Figure a, b, c and d are the representative images of GNP-C225 conjugates synthesized at ratio 0.76, 1.52, 2.29 and 3.76 respectively.
(TIF)

Figure S7 **Representative TEM images of tumor sections illustrating nanoconjugate location outside the tumor tissue.** GNP-IgG with the 1.5 ratio of Ab∶GNP are shown on the left and right (in a low magnification and high magnification, respectively) to illustrate the accumulation of the non-specific nanoconjugates outside of the tumor tissue.
(TIF)

Figure S8 ***In vivo*** **gold uptake in 5 vital organs determine by INAA.** *In vivo* uptake of GNP-C225 conjugates (at varying ratios of antibody) by vital organs; 24 hrs after the intraperitoneal injection of the conjugates into an orthotopic model of pancreatic cancer. The uptake was determined by measuring the gold concentration in the tumors by INAA. Y axis represents gold concentration as ppm.
(TIF)
