# Supplementary figures and images for: Designing Nanoconjugates to Effectively Target Pancreatic Cancer Cells In Vitro and In Vivo
Source: PLoS One. 2011 Jun 27;6(6):e20347. doi: 10.1371/journal.pone.0020347 (PMC3124468; doi:10.1371/journal.pone.0020347)

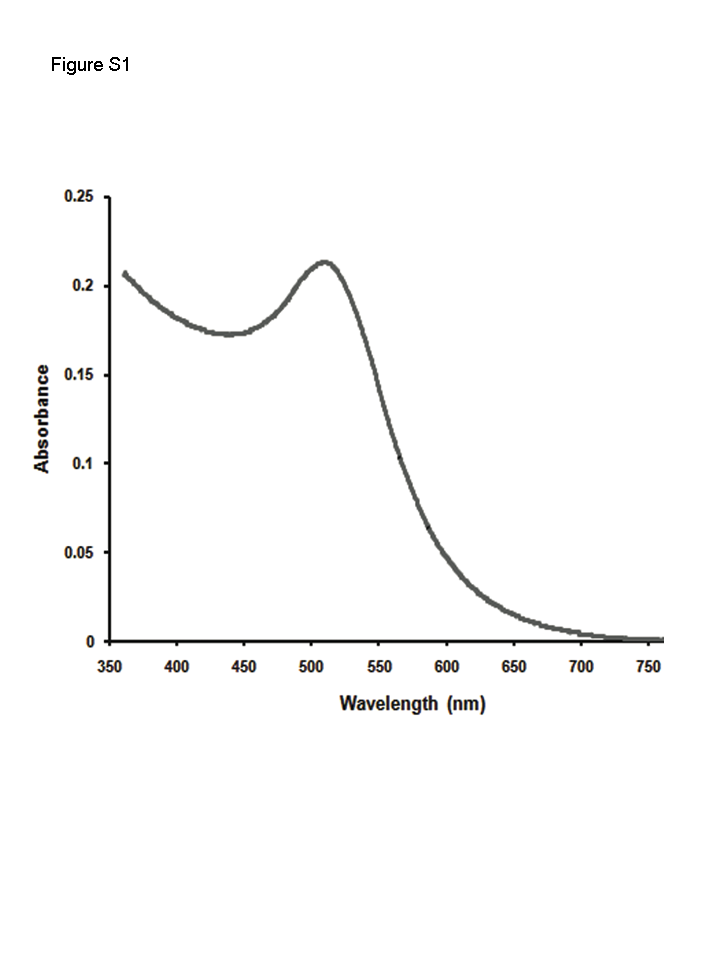

Supplement: Figure S1 — Representative absorption spectrum of the GNP used in the study. (TIF) [file pone.0020347.s001.tif]

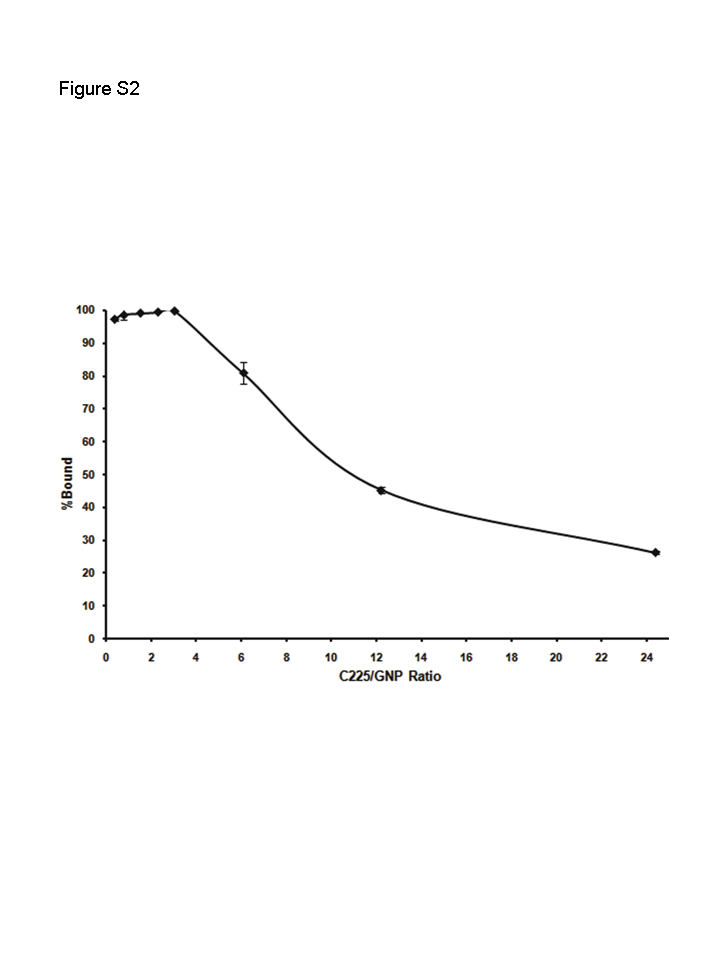

Supplement: Figure S2 — Binding of C225 to GNP determined by I125 labeled C225: Concentration of C225 bound to GNP with increasing concentration of C225 represented as the fraction of total C225 added. (TIF) [file pone.0020347.s002.tif]

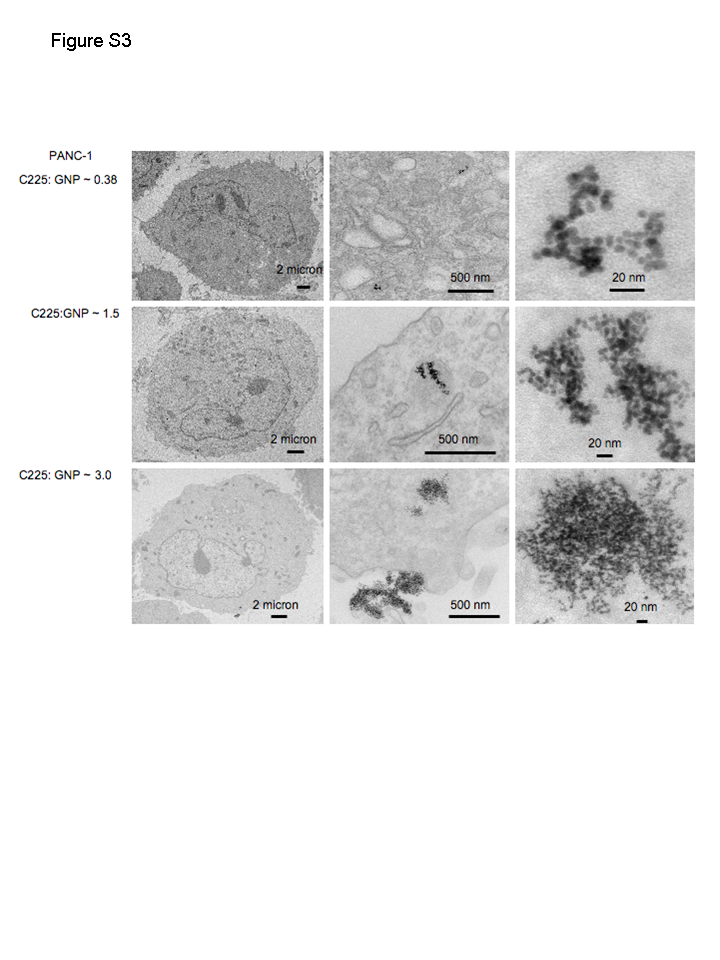

Supplement: Figure S3 — Internalization of different C225-GNP conjugates by Panc-1 Cells. Representative TEM images at different magnifications showing internalization of C225-GNP conjugates. (TIF) [file pone.0020347.s003.tif]

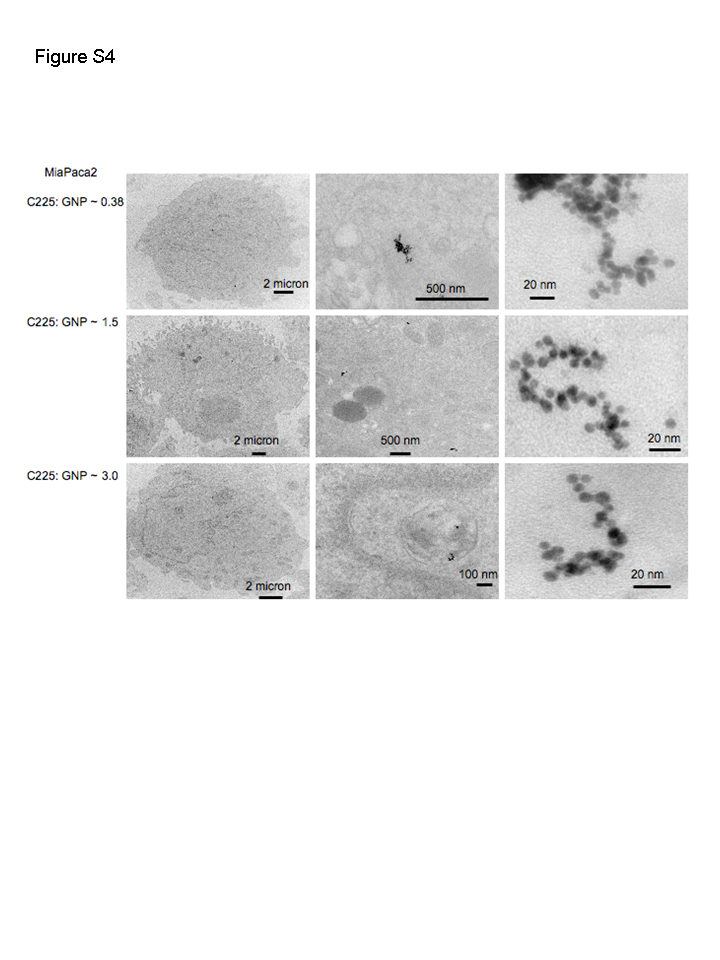

Supplement: Figure S4 — Internalization of different C225-GNP conjugates by MiaPaca-2 Cells. Representative TEM images at different magnifications showing internalization of C225-GNP conjugates. (TIF) [file pone.0020347.s004.tif]

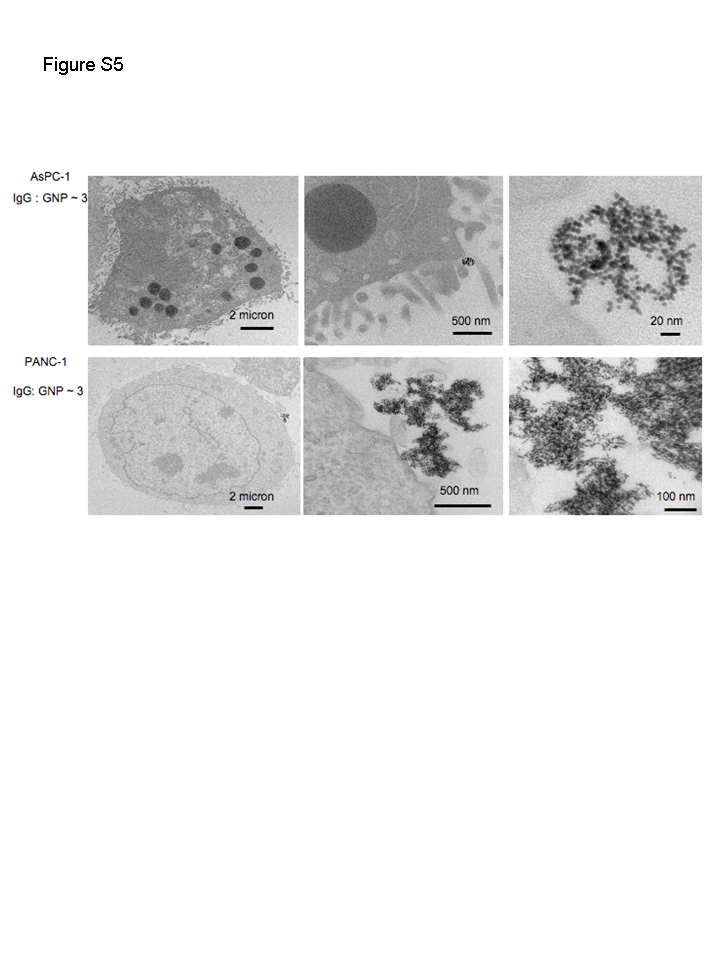

Supplement: Figure S5 — Internalization of GNP-IgG conjugates by PANC-1 and MiaPaca-2 Cells. Representative TEM images at different magnifications showing internalization of GNP-IgG conjugates. (TIF) [file pone.0020347.s005.tif]

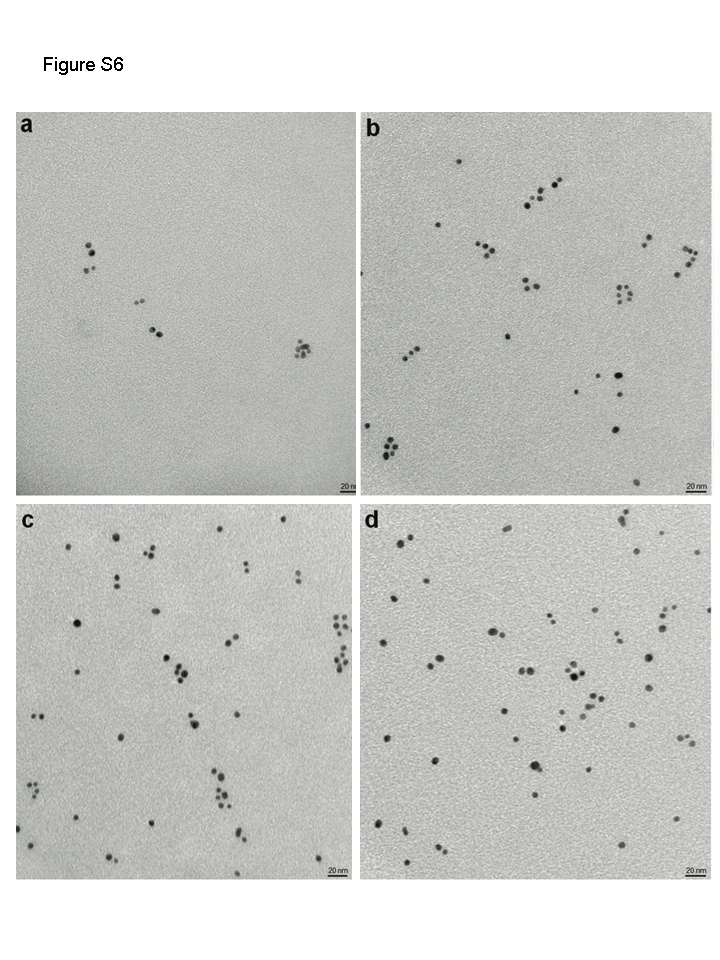

Supplement: Figure S6 — TEM images of GNP-C225 conjugates synthesized at different C225∶GNP ratio. Figure a, b, c and d are the representative images of GNP-C225 conjugates synthesized at ratio 0.76, 1.52, 2.29 and 3.76 respectively. (TIF) [file pone.0020347.s006.tif]

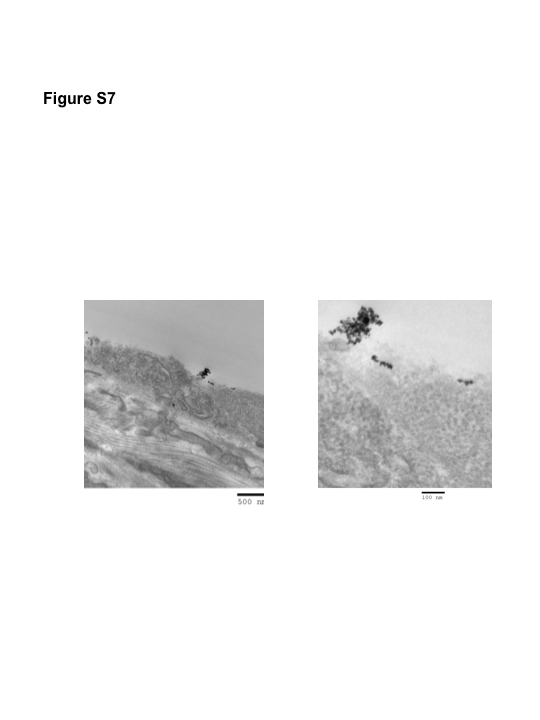

Supplement: Figure S7 — Representative TEM images of tumor sections illustrating nanoconjugate location outside the tumor tissue. GNP-IgG with the 1.5 ratio of Ab∶GNP are shown on the left and right (in a low magnification and high magnification, respectively) to illustrate the accumulation of the non-specific nanoconjugates outside of the tumor tissue. (TIF) [file pone.0020347.s007.tif]

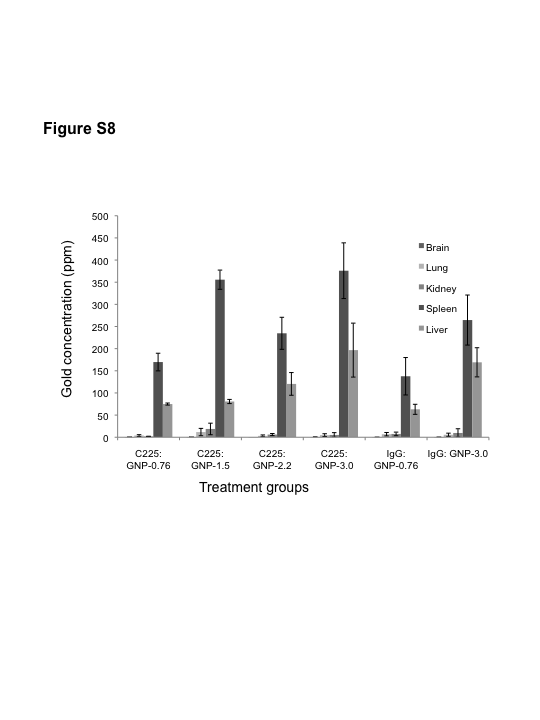

Supplement: Figure S8 — In vivo gold uptake in 5 vital organs determine by INAA. In vivo uptake of GNP-C225 conjugates (at varying ratios of antibody) by vital organs; 24 hrs after the intraperitoneal injection of the conjugates into an orthotopic model of pancreatic cancer. The uptake was determined by measuring the gold concentration in the tumors by INAA. Y axis represents gold concentration as ppm. (TIF) [file pone.0020347.s008.tif]
